# Insight into the Role of the miR-584 Family in Human Cancers

**DOI:** 10.3390/ijms25137448

**Published:** 2024-07-06

**Authors:** Mariantonia Braile, Neila Luciano, Davide Carlomagno, Giuliana Salvatore, Francesca Maria Orlandella

**Affiliations:** 1 IRCCS Synlab SDN, 80143 Naples, Italy; mariantonia.braile@synlab.it; 2 Dipartimento di Scienze Biomediche Avanzate, Università degli Studi di Napoli “Federico II”, 80131 Naples, Italy; neilaluciano14@gmail.com; 3 Dipartimento di Farmacia, Università degli Studi di Napoli “Federico II”, 80131 Naples, Italy; davide.carlomagno97@gmail.com; 4 CEINGE-Biotecnologie Avanzate Franco Salvatore, 80145 Naples, Italy; giuliana.salvatore@uniparthenope.it; 5 Dipartimento delle Scienze Mediche, Motorie e del Benessere, Università degli Studi di Napoli “Parthenope”, 80133 Naples, Italy

**Keywords:** cancer, microRNA, miR-584, target therapy, biomarker

## Abstract

Among the non-coding RNAs, the aberrant expression of microRNAs (miRNAs) is well described in the oncology field. It is clear that the altered expression of miRNAs is crucial for a variety of processes such as proliferation, apoptosis, motility, angiogenesis and metastasis insurgence. Considering these aspects, RNA-based therapies and the use of miRNAs as non-invasive biomarkers for early diagnosis are underlined as promising opportunities against cancer death. In the era of precision medicine, significant progress in next-generation sequencing (NGS) techniques has broadened knowledge regarding the miRNAs expression profile in cancer tissues and in the blood of cancer patients. In this scenario, pre-clinical and clinical studies suggested that the members of the miR-584 family, i.e., miR-584-5p and -3p, are prominent players in cancer development and progression. Under some conditions, these miRNAs are under-expressed in cancer tissues acting as tumor suppressors, while in other conditions, they are overexpressed, acting as oncogenes increasing the aggressive behavior of cancer cells. The aim of this review is to provide a comprehensive and up-to-date overview on the expression, upstream genes, molecular targets and signaling pathways influenced by the miR-584 family (i.e., miR-584-3p and -5p) in various human solid and hematological cancers. To achieve this goal, 64 articles on this topic are discussed. Among these articles, 55 are focused on miR-584-5p, and it is outlined how this miRNA could be used in future applications as a potential new therapeutic strategy and diagnostic tool.

## 1. Introduction

Small noncoding RNAs, known as microRNAs (miRNAs), are found in nearly every eukaryotic genome, including those of mammals as well as plants [1]. miRNAs control the expression of genes at the post-transcriptional level by their seed region composed of the first two to eight nucleotides at the 5′ and are able to identify similar sequences on the 3′ untranslated region (UTR) of the mRNA targets [2,3]. However, other non-canonical mechanisms were recently unveiled to be used by miRNA to find the expression of their target genes [4].

Numerous cellular processes, such as the immune response, cell motility, cell death, cell proliferation and response to treatment, are impacted by miRNAs during the development of several diseases [3]. In light of these, the role of miRNAs is abundantly studied in the oncology field, and, as a matter of fact, the aberrant miRNA expression is widely demonstrated in cancer [5]. From these studies, it emerged that the same miRNA appears to be downregulated in certain cancer types, where it acts as a tumor suppressor, while in other cancers, it is upregulated and acts as an oncogene. Hence, it is clear that the functions of these small molecules could depend on the cellular context and on the expression of their target mRNAs [6].

It is worth emphasizing that, as well as being tested as cancer therapeutic targets, the knowledge of miRNAs profiling in blood is underway, with the aim of using them as non-invasive biomarkers for early cancer detection and as a predictive factor of therapeutic responses [7].

There are several tools that have made it possible to unveil the miRNA expression profile in the tissues and liquid biopsy of cancer patients. In the last decades, besides q-RT-PCR and miRNA microarray hybridization, next-generation sequencing (NGS) allowed for the simultaneous detection of hundreds of miRNAs. Nonetheless, its high cost is one of its drawbacks. On the other hand, The Cancer Genome Atlas project (TCGA) project has considerably changed the approach regarding the molecular characterization of cancer tissues, representing an attractive resource to be used by easily interrogating several online tools [8].

Here, we focused on the miR-584 family, i.e., miR-584-3p and miR-584-5p (previous ID miR-584). These two miRNAs derive from a common precursor (pre-miR-584), encoded by a gene located on chromosome 5, which is processed into two different arms (-3p and -5p) in the cytoplasm.

In accordance with this, in this comprehensive review, we report the expression and the molecular pathways discovered to be altered by miR-584-3p and miR-584-5p with the aim of improving the knowledge regarding their potential application as biomarkers and as an effective target therapy for cancer patients.

To investigate the expression profile and biological mechanisms related to the members of the miR-584 family in human cancers, a comprehensive literature search was conducted interrogating the PubMed database by using the following keywords: “miR-584”, “miR-584-5p” and “miR-584-3p” in combination with “cancer”, “tumor” or “biomarker”. The search was stopped on 9 February 2024. 

From this research, we have excluded retracted articles or those for which the full text was not available. A total of 64 original articles are included in this review; 9 of them are focused on miR-584-3p, while 55 are focused on miR-584-5p. The articles are summarized in eight tables based on the issue covered, i.e., miR-584-5p expression in cancer tissues (Table 1, Table 2, Table 3, Table 4, Table 5 and Table 6), miR-584-3p expression in cancer tissues (Table 7) and miR-584-5p expression in peripheral blood (Table 8).

## 2. Unravelling the Role of miR-584-5p in Cancer Tissues and Cell Lines

### 2.1. Breast Cancer

Breast cancer (BC) is one of the most common malignancies in the world, representing approximately 32% of new cancer diagnoses in women, and it is the second leading cause of cancer death [9]. miR-584-5p expression and its targets genes in BC are defined in several papers (Table 1).

The first paper by Fils-Aimé and colleagues unveils that the expression of miR-584-5p is downregulated and under the control of TGF-β, an important player of tumor progression. Indeed, upon the downregulation of miR-584-5p, induced by TGF-β, there occurred an overexpression of the PHACTR1 and a rearrangement of the actin cytoskeleton, stimulating the migration capability of BC cell lines [10]. The protein phosphatase PHACTR1 is an actin binding protein that regulates the reorganization of the actin cytoskeleton [11].

**Table 1:** Summary of target genes and signaling pathways correlated to miR-584-5p downregulation in cancer tissues and cell lines.

Cancer Type	Expression	Sample (Patients, *n*)	Technique	Regulator	Validated Target	Functional Studies	Years	Ref.
BC	Down	Cell lines	q-RT-PCR	TGF-β	PHACTR1	↓ Motility *	2013	[10]
	Down	Cell lines	RNA-seq	AnAc	ns	ns	2017	[12]
	DEG	Tissues (*n* = 57)	In silico (GEO database)	Paclitaxel	ns	ns	2021	[13]
GC	Down	Tissues (*n* = 75) Cell lines	q-RT-PCR	Ns	WWP1	↓ Proliferation * ↑ Senescence * ↑ Apoptosis * ↓ In vivo tumorigenesis *	2017	[14]
	Up (early stage) Down (late stage)	Tissues (*n* = 37)	q-RT-PCR/ In silico (TCGA)	*H. pylori* infection	ns	ns	2020	[15]
Osteo-sarcoma	Down	Tissues (*n* = 37) Cell lines	q-RT-PCR	Ns	CCN2/CTGF	↓ Proliferation * ↓ Motility * ↑ Apoptosis * ↓ Drug resistance *	2020	[16]
	Down	Cell lines	q-RT-PCR	Ns	ns	↓ Proliferation * ↓ Motility * ↑ Apoptosis *	2023	[17]
	Down	Tissues (*n* = 24)	q-RT-PCR	Ns	ns	ns	2019	[18]
Ewing sarcoma	Down	Tissues (*n* = 19)	q-RT-PCR	Ns	ns	ns	2020	[19]
Cervical cancer	Down	Tissues (*n* = 30) Cell lines	q-RT-PCR	Ns	GLI1	↓ Proliferation * ↓ Motility * ↓ Drug resistance *	2020	[20]
ccRCC	Down	Tissues (*n* = 14) Cell lines	miRNA array/ q-RT-PCR	Ns	ROCK1	↓ Motility *	2011	[21]
HNSCC	Down	Tissues (*n* = 352)	ISH technology	Ns	SQLE	↓ Proliferation * ↓ Motility *	2022	[22]
Bladder cancer	Down	Tissues (*n* = 60) Cell lines	q-RT-PCR	circ-LONP2	YAP1	↑ Proliferation ↓ Apoptosis ↑ Invasion	2022	[23]

AnAc: anacardic acid; BC: breast cancer; ccRCC: clear cell renal carcinoma: DEG: differentially expressed genes; ISH: in situ hybridization; GC: gastric cancer; GEO: Gene Expression Omnibus database; HNSCC: head and neck squamous cell carcinoma; ns: not specified. * Effects following the treatment with the miR-584-5p mimic.

Interestingly, from whole genome expression profiling, it emerged that another possible regulator of miR-584-5p in BC is anacardic acid (AnAc), an important lipid that exerts anticancer activity by inhibiting the proliferation of BC cells [12]. 

Lastly, consulting the GSE114403 dataset from the Gene Expression Omnibus (GEO) database, it was found that miR-584-5p is among deregulated miRNAs in the BC tissues of 50 women after paclitaxel treatment compared to 50 pre-treatment BC samples, suggesting the potential role of miR-584-5p in the molecular mechanisms influenced by this drug [13].

### 2.2. Gastric Cancer

A worldwide healthcare challenge is gastric cancer (GC). The prognosis for these patients remains poor, even with improved chemotherapy and surgical options [24]. 

Two studies suggested that miR-584-5p could behave as a tumor suppressor in GC, suggesting its potential employment as a target therapy and biomarker for GC patients (Table 1). 

For the first time, the downregulation of miR-584-5p was reported in GC tissues and cell lines by Li and colleagues [14]. The mimic of miR-584-5p suppressed cell proliferation and caused apoptosis and senescence by direct binding to the 3′UTR of the ubiquitin protein ligase WWP1, a protein that acts as an oncogene in several human cancers, including GC [25]. Moreover, the authors found that miR-584- 5p/WWP1 promoted the TGF-β pathway and also inhibited the tumorigenicity in a xenograft mice model of GC [14]. 

A recent work confirmed lower miR-584-5p expression in metastases and in late stages of GC; in spite of that, its dysregulation is complex, as miR-584-5p expression is higher in early stages of GC and in patients with *Helicobacter pylori* infection, suggesting a fluctuating miR-584-5p expression with GC progression [15]. The in silico analysis showed an inverse correlation between miR-584-5p and the transcription factor STAT1 in GC tissues deposited in TCGA; however, the luciferase assay is missing in order to confirm the direct targeting [15]. Overall, these data suggested the potential application of miR-584-5p as a diagnostic and prognostic biomarker in GC.

### 2.3. Bone Cancer

Osteosarcoma is the most frequent primary malignant bone tumor involving multiple potential genetic drivers [26].

Li and colleagues revealed that miR-584-5p is downregulated in osteosarcoma tissues compared to non-neoplastic bone and in osteosarcoma cell lines affecting the prognosis and the sensitivity to cisplatin and taxanes, respectively [16] (Table 1). 

Mechanistically, the forced expression of miR-584-5p hindered the proliferation and motility of osteosarcoma cell lines by directly targeting the 3′UTR of CCN2 (also known as CTGF) (Table 1) [16]. Moreover, the tumor suppressor function of miR-584-5p and potential CCN2 targeting was also suggested by Lu and colleagues (Table 1) [17]. Interestingly, the target gene is a member of the cellular communication network (CCN) family involved in the tumor microenvironment and in the progression of several malignant diseases, suggesting its role as potential target therapy [27,28].

Notably, a reduction in miR-584-5p has also been reported in childhood osteosarcoma, although it is not statistically significant, maybe due to the variable miR-584-5p distribution among the biopsies [18] (Table 1).

Also in Ewing sarcoma, a common pediatric bone cancer, the low levels of miR-584-5p are correlated with tumor progression and relapse after treatment, suggesting its potential application as a prognostic biomarker [19] (Table 1). 

### 2.4. Thyroid Cancer

Thyroid cancer (TC) is the most common endocrine tumor, which includes different subtypes ranging from differentiated, generally with a good survival rate (greater than 98%), to undifferentiated TC (or anaplastic, ATC), which is the most aggressive form, with a median overall survival (OS) of only 4 months [9,29].

In ATC tissues, the TWIST1 transcriptional factor directly binds a region upstream of miR-584-5p, inducing its overexpression. The overexpression of miR-584-5p induced resistance to apoptosis by directly targeting the 3′UTR of TUSC2 mRNA (also known as FUS1), an important tumor suppressor downregulated in several human cancers [30] (Table 2). In accordance with the tumor suppression function of TUSC2 in other cancers, in a subsequent paper, it was shown that in TC, this gene hindered cell proliferation and motility and increased sensitivity to apoptosis by stimulating SMAC/DIABLO and CYTOCHROME C proteins [31].

**Table 2:** Summary of target genes and signaling pathways correlated with miR-584-5p overexpression in cancer tissues and cell lines.

Cancer Type	Expression	Sample (Patients, *n*)	Technique	Regulator	Validated Target	Functional Studies	Years	Ref.
TC	Up (ATC)	Tissues (*n* = 27)	q-RT-PCR	TWIST1	TUSC2	↑ Drug resistance	2016	[30]
	Up	Tissues (*n* = 104)	miRNA array	ns	ns	ns	2012	[32]
	Up (PTC)	Cell lines	q-RT-PCR	ns	ns	↓ Motility	2015	[33]
Mesothe-lioma	Up	Tissues (*n* = 17)	miRNA array	ns	ns	ns	2009	[34]
AML	Up	Kasumi-1 cell line	miRNA array/ q-RT-PCR	CD34^+^38^−^	ns	ns	2010	[35]

AML: acute myeloblastic leukemia; ATC: anaplastic thyroid cancer; ns: not specified; PTC: papillary thyroid cancer; TC: thyroid cancer.

Consistent with these findings, the high expression of miR-584-5p also emerged from a miRNA array carried out on TC tissues compared to benign thyroid lesions [32] (Table 2). 

Moreover, miR-584-5p was found to also be upregulated in papillary thyroid cancer cell lines compared to normal thyroid tissues, although its forced expression reduces the cell migration ability by attenuating the expression of the oncogenic kinase ROCK1 [33] (Table 2). ROCK1 is known to be a downstream effector of the RhoA signaling pathway that influences the behavior of cancer cells modulating cell motility and the epithelial-to-mesenchymal transition [36,37]. Interestingly, in TC, other miRNAs (i.e., miR-421 and miR-26a) were recently reported to be negative regulators of the mRNA of ROCK1, suggesting the importance of the 3′UTR in the expression level of this gene during tumorigenesis [38,39]. 

### 2.5. Colorectal Cancer

Colorectal cancer (CRC) represents the third leading cause of cancer death [9,40]. Indeed, despite the screening and the increase in knowledge regarding genetic and environmental factors and the molecular pathways altered in CRC patients, there is a need to uncover novel methods for early diagnosis and to identify novel potential therapeutic agents. 

As reported in Table 3, the upregulation of this miRNA was revealed by Gaedcke and colleagues through a miRNA microarray performed on 57 rectal cancer tissues compared to matched mucosa samples [41]. Wang and colleagues reported that a downregulation of miR-584-5p occurred in the CRC cell line (HT-29) after the transfection of NGX6, a tumor suppressor gene that, through bioinformatic analyses, the authors also found to be associated with the regulation of apoptosis and motility and to be involved in the JNK and NOTCH pathways [42].

**Table 3:** Summary of the controversial role of miR-584-5p in human cancer.

Cancer Type	Expression	Sample (Patients, *n*)	Technique	Regulator	Validated Target	Functional Studies	Years	Ref.
CRC	Up	Tissues (*n* = 57)	miRNA array	ns	ns	ns	2012	[41]
	Down	NGX6-transfected cells	miRNA array/ q-RT-PCR	NGX6	ns	ns	2010	[42]
	Up	Cell lines	dd-RT-PCR	Anti miR-584-5p-PNA	ns	↓ Proliferation * ↑ Apoptosis *	2022	[43]
	Up	Cell line and exosomes after treatment with FF/cAP18	q-RT-PCR	FF/CAP18 (antimicrobial peptide)	ns	ns	2018	[44]
PC	Down	Tissues (*n* = 26) Cell lines	q-RT-PCR	ns	CCND1	↓ Invasion * ↓ Proliferation *	2019	[45]
	Up	Gemcitabine-resistant PC cells	In silico (GEO database)/ q-RT-PCR/	Gemcitabine	ns	↑ Drug resistance *	2020	[46]
EC	Down	Tissues (*n* = 24)	q-RT-PCR	ns	ns	ns	2022	[47]
	Up	Tissues (*n* = 12 in q-RT-PCR, *n* = 9 in TCGA)	q-RT-PCR/ In silico (TCGA)	ns	ns	ns	2017	[48]

CRC: colorectal cancer; dd-RT-PCR: droplet digital-RT-PCR; EC: esophageal cancer; EAC: esophageal adenocarcinoma; ESCC: esophageal squamous cell carcinoma; GEO: Gene Expression Omnibus database; ns: not specified; PC: pancreatic cancer; PNA: peptide nucleic acids. * Effects following the treatment with PNA [43] or with miR-584-5p mimic [45,46].

The expression of miR-584-5p was investigated in CRC cell lines (Table 3) and in blood (Table 8) from CRC patients by Gasparello and colleagues. After unveiling the upregulation of miR-584-5p in the liquid biopsy of early-stage CRC patients (Table 8) [49], the authors showed that this miRNA is upregulated in CRC cell lines, where it exerts oncogenic activity (Table 3) [43]. Indeed, in this experimental set, anti-miR-584-5p nucleic acids functionalized with an R8 peptide (R8-PNA-a584) were used to demonstrate that the selective downregulation of miR-584-5p inhibited cell proliferation and increased apoptosis. At last, an increase in the percentage of apoptotic cells was detected by the combined treatment of sulforaphane and R8-PNA-a584, suggesting that this strategy could be used to fight CRC cancer [43]. 

Contrariwise, Hayashi and colleagues found an increased expression of miR-584-5p in the CRC cell line (HCT116) and in their exosome after treatment with the antimicrobial peptide FF/CAP18 (Table 3) [44]. Moreover, since FF/CAP18 treatment inhibits cell proliferation in this system, the authors suggested that this suppressive effect could be mediated by the upregulation of miR-584-5p [44]. 

### 2.6. Lung Cancer

This section highlights the emerging interest regarding the potential role of miR-584-5p as a target therapy in lung cancer (LC) since 10 articles emerged from our research and are reported in Table 4.

Briefly, LC is one of the most common and deadly types of cancer worldwide [9]. Non-small cell lung cancer (NSCLC), constituting approximately 85% of all LC cases, encompasses three primary histological subtypes: lung adenocarcinoma (LUAD), the most prevalent subtype; lung squamous cell carcinoma (LUSC); and large cell carcinoma.

The possible employment of miR-584-5p as a prognostic biomarker in the LUAD subtype was investigated in several studies by interrogating the TCGA database [50,51,52,53] (Table 4). This research revealed that, despite the downregulation of miR-584-5p in LUAD tissues deposited in TCGA [50,51], the higher expression of this miRNA appears to be associated with a worse OS in LUAD patients, representing a potential risk factor [50,52,53]. Finally, Shan and colleagues found that the circulating level of miR-584-5p is higher in LUAD patients (Table 8), while no differences were found in 24 LUAD cancer tissues compared to adjacent normal tissues from patients enrolled in the study [54].

**Table 4:** Summary of the role of miR-584-5p in NSCLC cancer.

Expression	Sample (Patients, *n*)	Technique	Regulator	Validated Target	Functional Studies	Years	Ref.
Down	LUAD tissues (*n* = 567)	In silico (TCGA)	ns	ns	ns	2019	[50]
Down	LUAD tissues (*n* = 494)	In silico (TCGA)	ns	ns	ns	2021	[51]
Up *	LUAD tissues (*n* = 479)	In silico (TCGA)	ns	ns	ns	2019	[52]
Up *	LUAD tissues (*n* = 76 for in silico; *n* = 8 for miRNA seq.)	In silico (TCGA)/ miRNA sequencing	ns	ns	ns	2023	[53]
Nc	LUAD tissues (*n* = 24)	q-RT-PCR	ns	ns	ns	2020	[54]
Down	Tissues (*n* = 626)	In silico (GEO database)	ns	ns	ns	2016	[55]
Down	Tissues (*n* = 63) Cell lines	q-RT-PCR	circ_0079530	THBS2	ns	2023	[56]
Down	Cell lines	q-RT-PCR	ns	YKT6	↓ Motility ** ↓ In vivo tumorigenesis **	2021	[57]
Up	Tissues treated with taxane (*n* = 874) Cell lines	miRNA array	SNP rs2662411	ns	ns	2012	[58]
Down	Tissues (*n* = 30) Cell lines	q-RT-PCR	ns	MMP-14	↓ Motility **	2019	[59]

* Cox regression analyses. ** Effects following the ectopic expression of miR-584-5p. GEO: Gene Expression Omnibus database; LUAD: lung adenocarcinoma; SNP: single-nucleotide polymorphisms; ns: not specified; NSCLC: non-small cell lung cancer.

Regardless of the tumor subtype, the downregulation of miR-584-5p in NSCLC patients was also confirmed by the comparative study of Hu and colleagues, in which five independent microarray datasets were analyzed [55], and by the study of Fang and colleagues conducted on 30 cancer and matched normal tissues from NSCLC patients [56].

Mechanistically, one of the possible mechanisms of miR-584-5p deregulation in LC patients is through its binding to circular RNA (circRNA). Indeed, circ_0079530 was identified as a potential negative regulator of this miRNA in LC. Resultantly, circ_0079530, by sponging miR-584-5p, contributed to the progression of NSCLC [56].

Another possible mechanism of miR-584-5p deregulation in this tumor could be through epigenetic factors. Indeed, experiments on LC cell lines and on an animal model conducted by Lee and colleagues suggested that the methylation of the miR-584-5p promoter induced a downregulation of this miRNA and is positively correlated with the progression of LC stages [57]. 

In the work of Niu and colleagues, a genome-wide association study (GWAS) was conducted in human lymphoblastoid cell lines in order to identify possible single-nucleotide polymorphisms (SNPs) correlated with paclitaxel chemotherapy responses in LC patients. The GWAS analysis, followed by genotyping studies in the DNA of 76 small cell lung cancer (SCLC) and in 798 NSCLC patients treated with paclitaxel, revealed that the SNPs rs2662411 was associated with OS and with higher miR-584-5p expression [58]. 

Regarding the biological effects and molecular signaling pathways induced by miR-584-5p in LC, functional experiments unveiled that its ectopic expression inhibited the migration and invasion of NSCLC cells by directly targeting YKT6 [57] and MMP-14 [59]. The importance of miR-584-5p target genes in the progression of NSCLC tumors is supported by several works; for instance, higher levels of YKT6 [60] (a protein involved in the production and release of exosome) and of MMP-14 (an important Matrix metalloproteinase) [61] are correlated with the shorter overall survival of these patients.

Additionally, miR-584-5p negatively modulates the cell proliferation, motility and metastasis of NSCLC through the regulation of THBS2 expression, a glycoprotein upregulated in NSCLC [56]. THBS2 was also recently found to be overexpressed in NSCLC tissues compared to adjacent tissues, where it is correlated with a poor prognosis [62].

Taken together, these data suggest that miR-584-5p is a potential prognostic factor and target therapy in LC progression. 

### 2.7. Pancreatic Cancer

Significant efforts are attempting to define the major genetic factors driving the pathogenesis and progression of pancreatic cancer (PC) since it is one of the deadliest cancers in the world [63]. Even in this malignancy, the role of miR-584-5p needs to be clarified since there are few and conflicting data (Table 3).

In the first study, conducted by Chen and colleagues, it has been demonstrated that the expression of miR-584-5p is significantly downregulated in the primary cancer tissues of PC patients who had not undergone any treatment before surgical resection [45]. The authors also confirmed this downregulation in PC cell lines, demonstrating that miR-584-5p restoration reduces cell proliferation and invasion by directly targeting the 3‘UTR of CCND1 mRNA, an important regulator of the cell cycle, responsible for the transition of G1 to the S phase [45]. Recent studies revealed that the higher level of CCND1 in PC tissues represents a negative prognostic factor for these patients and could also be due to the actions of other miRNAs such as miR-503 [64] and miR-497-5p [65].

Additionally, the role of miR-584-5p in gemcitabine-resistant PC cells and its correlation with immune infiltration were clarified by Gu and colleagues [46]. The authors, querying the GEO dataset, unveiled that gemcitabine resistance modulated the CD4+ memory T cell fraction and altered the expression of miR-584-5p and its predicted target, YKT6. Moreover, Kaplan–Meier survival curves showed that high level of miR-584-5p is associated with a worse prognosis of PC patients [46]. 

### 2.8. Hepatocellular Carcinoma

Hepatocellular carcinoma (HCC) is a deadly tumor characterized by a poor outcome, as patients show resistance to therapeutic treatments [66]. The aberrant expression of miR-584-5p is well described in HCC tissue (Table 5).

In Song’s paper, it was demonstrated that the downregulation of miR-584-5p occurred in HCC tissues and is associated with cancer aggressiveness (TNM stage, lymph node metastasis and tumor size) [67]. Moreover, in HCC cells lines, miR-584-5p restoration reduced proliferation and invasion through the direct target BDNF, a known oncogene involved in the genesis and progression of HCC [67]. Indeed, miR-584-5p expression was inversely correlated with BDNF in HCC tissues [67].

**Table 5:** Summary of the role of miR-584-5p in HCC.

Expression	Sample (Patients, *n*)	Technique	Regulator	Validated Target	Functional Study	Years	Ref.
Down	Tissues (*n* = 56) Cell lines	q-RT-PCR	ns	BDNF	↓ Proliferation * ↓ Motility *	2019	[67]
Down	Tissues (*n* = 3) Cell lines	In silico (GEO database) q-RT-PCR	Circ_1306	CDK16	↓ Proliferation ↓ In vivo tumorigenesis	2020	[68]
Down	DUXAP8 overexpressing cell lines Mice	q-RT-PCR	lncDUXAP8	MAPK1	↓ Motility * ↑ Chemosensitivity *	2021	[69]
Up	Cell lines	q-RT-PCR	ns	KCNE2	↑ Proliferation ↑ Motility	2019	[70]
Down	Tissues (*n* = 1224)	In silico (TCGA and GEO database)	ns	ns	ns	2018	[71]
Up	Cell line and Exosome	q-RT-PCR	ns	PCK1	↑ Proliferation ↑ Migration ↑ Angiogenesis	2020	[72]
Up	Tissues (*n* = 40) Cell lines	q-RT-PCR	CircSMYD4	ns	↑ Proliferation ↑ Migration ↑ In vivo tumorigenesis	2020	[73]

GEO: Gene Expression Omnibus database; ns: not specified. * Effects following ectopic expression of miR-584-5p.

The downregulation of miR-584-5p in HCC could be due to the alteration of various possible factors, including circRNAs and long noncoding RNAs (lncRNAs). The circ_1306, overexpressed in HCC tissues and cell lines, regulated the expression of miR-584-5p and, consequently, its direct target CDK16, promoting tumorigenesis both in vitro and in vivo [68]. Likewise, the lncRNA DUXAP8, overexpressed in the HCC tissues taken from tumor-bearing mice resistant to Sorafenib, exerts its oncogenic activity by sponging the miR-584-5p through a competitive endogenous RNA (ceRNA) mechanism, thus modulating the MAPK/ERK axis [69].

In contrast to previous works, other studies outlined the role of this miRNA as an oncomiR in HCC. Wei and colleagues proposed that miR-584-5p functions as an oncogenic miRNA by targeting KCNE2. Indeed, in vitro experiments showed that the miR-584-5p silencing increased cell proliferation and motility [70]. Finally, Kaplan–Meier plotter analysis confirmed that the high level of this miRNA is correlated with the OS of HCC patients [70].

The potential prognostic power of miR-584-5p in HCC was investigated by Nagy and colleagues by consulting the data of miRNA-seq deposited in TCGA and the microarray data in GEO datasets [71]. This survival analysis showed that a low level of miR-584-5p in tumor tissues is significantly correlated with the OS of HCC patients [71].

In the work of Shao, miR-584-5p was overexpressed in HCC cells and in vesicles released by these cells. This overexpression activated the NRF2 pathway and promoted the aggressive phenotype of HCC cells by directly targeting the mRNA of PCK1. According to the in vitro results, in vivo experiments showed that miR-584-5p forced expression enhanced tumor growth and the angiogenic process [72]. 

Another study showed that the circSMYD4 is downregulated in HCC tissues and cell lines, and its restoration impaired the aggressive behavior of HCC cells and inhibited tumor growth in vivo by sponging miR-584-5p [73]. 

As is evident from these articles, the actual role of this miRNA in HCC is not yet clear.

### 2.9. Esophageal Cancer 

Esophageal cancer (EC), based on molecular and clinical characteristics, is classified in esophageal adenocarcinoma (EAC) and in esophageal squamous cell carcinoma (ESCC), both characterized by high aggressiveness and a significant mortality rate [74].

As reported in Table 3, miR-584-5p behaves as a potential biomarker in the screening of EAC and for the early detection of ESCC, but with a different trend.

Indeed, through miRNA expression profiling, it was discovered that miR-584-5p is one of the 12 miRNAs downregulated in the biopsies of patients with Barrett’s syndrome who progressed to EAC compared to the group of patients in whom this syndrome did not assume an aggressive phenotype [47]. Meanwhile, through an investigation carried out by q-RT-PCR tests and by in silico analysis on the TCGA database, an overexpression of miR-584-5p was found in the plasma and in cancer tissues extracted from ESCC patients. These results suggest the potential role of miR-584-5p as a non-invasive biomarker for the early diagnosis of ESCC [48].

### 2.10. Brain Cancer 

Various studies focused on the role of miR-584-5p within brain tumors (Table 6). Gliomas represent the predominant type of primary brain tumors, constituting over 70% of all primary neoplasms within the central nervous system. They exhibit significant diversity in terms of morphology, location, genetic mutations and treatment responsiveness [75].

Concerning gliomas tumorigenesis, miR-584-5p is implicated in both development and progression. Indeed, the dysregulation of miR-584-5p was found in both glial tumors and glioma cell lines where it acts as a tumor suppressor by targeting the PTTG1IP, a protein associated with tumor growth [76].

**Table 6:** Summary of the role of miR-584-5p in brain cancers.

Cancer Type	Expression	Sample (Patients, *n*)	Technique	Regulator	Validated Target	Functional Studies	Years	Ref.
Glioma	Down	Tissues (*n* = 13) Cell lines	q-RT-PCR	ns	PTTG1IP	↓ Proliferation *	2014	[76]
Pediatric glioma	Up	Tissues (*n* = 120)	q-RT-PCR	ns	ns	ns	2018	[77]
GBM	Down	Tissues (*n* = 58) Cell lines	q-RT-PCR	circPITX1	KPNB1	↓ Proliferation * ↓ Motility * ↓ Angiogenesis *	2021	[78]
NB	Down	Tissues (*n* = 43) Cell lines	q-RT-PCR	LINC01296	TRIM59	↑ Proliferation ↑ Motility	2022	[79]
	Down	Cell lines	q-RT-PCR	LINC0196	TRIM59	↓ Proliferation * ↓ Apoptosis * ↓ Motility *	2022	[80]
MB	Down	Mice/ Cell lines	q-RT-PCR	ns	HDAC1/ eIF4E3	↓ Proliferation * ↓ Drug resistance *	2018	[81]

GBM: glioblastoma; MB: medulloblastoma; NB: neuroblastoma; ns: not specified. * Effects following the ectopic expression of miR-584-5p.

Contrariwise, in pediatric patients with low-grade glioma, the overexpression of miR-584-5p was found by Tantawy and colleagues [77].

The role of miR-584-5p was unveiled in glioblastoma (GBM), a highly aggressive and malignant form of glioma classified as grade IV in the WHO classification [82]. In the study by Cao and colleagues, circPITX1 promoted carcinogenesis by sequestering miR-584-5p and by upregulating its target, KPNB1 [78].

Neuroblastoma (NB), a malignancy arising from immature nerve cells during embryonic development, frequently occurs near the adrenal glands and is one of the prevalent solid tumors in early childhood [83]. In this cancer, the downregulation of miR-584-5p is due to two lncRNAs, LINC01296 [79] and LINC0196 [80], and caused an overexpression of the mRNA of TRIM59. 

In medulloblastoma (MB), an embryonal neuroepithelial tumor primarily affecting the cerebellum, miR-584-5p was found to be downregulated [81]. Its restoration in MB cell lines resulted in a higher response to vincristine treatment and in a higher response to radiation by inducing cell cycle arrest and apoptosis. These results suggest a tumor suppression function of miR-584-5p in MB. Mechanistically, the effects mediated by miR-584-5p occur by targeting the mRNA of eIF4E3 and HDAC1 [81].

The conflicting findings across the studies underscore the need for further investigations to elucidate the precise role of miR-584-5p in brain pathogenesis and its potential implications for therapeutic strategies.

### 2.11. Other Cancers

The importance of miR-584-5p also emerged in other malignancies, where it influences a variety of biological processes, acting as a tumor suppressor (Table 1) or as an oncogene (Table 2). Indeed, miR-584-5p exerts tumor suppressor properties in cervical carcinoma [20] and in clear cell renal carcinoma (ccRCC) tissues [21], impairing the expression of GLI1 and of ROCK1, respectively (Table 1). 

Recently, it was reported that the lower expression of miR-584-5p in head and neck squamous cell carcinoma (HNSCC) compared to paratumor oral mucosa is responsible for the upregulation of SQLE, an enzyme involved in cholesterol synthesis. Indeed, the authors found that SQLE upregulation is correlated with an advanced stage, metastasis and a worse prognosis [22]. Mechanistically, the upregulation of SQLE in a miR-584-5p-dependent manner, boosting the PI3K/AKT signaling pathway, promotes cell proliferation and cell motility in HNSCC cells [22] (Table 1).

A significantly lower expression of miR-584-5p also emerged in bladder cancer tissues compared to paired normal tissues, where it correlated with a worse OS [23]. Mechanistically, miR-584-5p downregulation appeared to be under the control of the circular RNA circLONP2 and responsible for YAP1 overexpression [23] (Table 1).

In order to investigate the miRNAs signature in malignant mesothelioma, a miRNA array was carried out by Guled and colleagues [34]. This analysis revealed that miR-584-5p is overexpressed in this cancer compared to paired normal tissue samples (Table 2).

Through a microarray containing 723 human miRNAs, the miRNoma of the acute myeloblastic leukemia (AML) Kasumi-1 cell line was also investigated [35]. This cell line is characterized by a particular subpopulation of CD34^+^38^−^ cells that are considered the leukemia-initiating cells [84]. The miRNA expression profile of Kasumi-1 cells revealed that miR-584-5p and miR-182 are miRNAs upregulated in the CD34^+^38^−^ subpopulation compared to the other Kasumi-1 subpopulations (CD34^−^ and CD34^+^38^+^), making these two miRNAs a faithful tool for highlighting the early myeloid stem cells [35]. (Table 2). The authors, through an in silico analysis, found that among the miR-584-5p predicted targets, there are genes involved in hematopoietic cell proliferation and differentiation and in the regulation of WNT and p53 signaling pathways, supporting the role of miR-584-5p in the development of AML [35]. 

Notably, the expression of miR-584-5p was not changed in CD34^+^ marrow cells isolated from myelodysplastic syndrome patients before and after in vitro lenalidomide treatment [85]. 

## 3. Unravelling the Role of miR-584-3p in Cancer Tissues and Cell Lines

The expression profile of miR-584-3p has been defined in a few tumors, in which it resulted to be, predominantly, downregulated (Table 7).

Interestingly, an increasing amount of evidence has been provided on the role of lncRNAs as regulators of miR-584-3p expression in cancers. The high lncRNA LOC101927746 expression, associated with the tumor stage and metastasis in CRC patients, induced an inhibition of the miR-584-3p activity and, as a consequence, the overexpression of its predicted target SSRP1, a gene involved in the regulation of gene expression [86].

**Table 7:** Summary of the role of miR-584-3p in human cancer tissues.

Cancer Type	Expression	Sample (Patients, *n*)	Technique	Regulator	Validated Targets	Functional Studies	Years	Ref.
CRC	Down	Tissues (*n* = 25)	q-RT-PCR	lncRNA LOC101927746	SSRP1	↑ Proliferation ↑ Motility	2019	[86]
HCC	Down	Cell lines	q-RT-PCR	lncRNA LINC00607	ROCK1	↑ Proliferation ↑ Motility	2023	[87]
Glioma	Down	Tissues (*n* = 26)	q-RT-PCR	hypoxic condition	ROCK1	↓ Motility	2016	[88]
	Down	Tissues (*n* = 84)	q-RT-PCR	ns	ns	↓ Vasculogenic mimicry	2017	[89]
LC	Down	Tissues (*n* = 974)	In silico (GDC database)	ns	ns	ns	2022	[90]
	Down	Tissues (*n* = 60) Cell lines	q-RT-PCR	lncRNA LINC00174	RPS24	↓ Proliferation * ↓ Motility *	2022	[91]
	Down	Tissues (*n* = ns)	In silico (TGCA)/ q-RT-PCR	lncRNA TFAP2A-AS1	CDK4	↓ Proliferation * ↓ Motility *	2021	[92]
GC	Down	Tissues (*n* = 60) Cell lines	q-RT-PCR	ns	MMP-14	↓ Proliferation * ↓ Angiogenesis * ↓ In vivo tumorigenesis and metastasis *	2017	[93]
Melanoma	Up	Metformin-treated cells	NGS	Metformin	SCAMP3	↓ Proliferation * ↑ Apoptosis *	2019	[94]

CRC: colorectal cancer; GC: gastric cancer; GDC: Global Data Center; HCC: hepatocellular carcinoma; LC: lung cancer; NGS: next-generation sequencing; ns: not specified. * Effects following ectopic expression of miR-584-5p.

In HCC too, the MYC-induced enhanced expression of lncRNA LINC00607 promoted cell motility and proliferation by controlling the miR-584-3p/ROCK1 axis [87]. 

Additionally, in glioma, miR-584-3p acts as a tumor suppressor impairing cell motility by regulating ROCK1 [88,89]. Furthermore, higher levels of miR-584-3p were found in patients with late-stage glioma that have a prolonged survival time, suggesting its role as a prognostic biomarker and target therapy [88].

In LC, the lncRNA LINC00174 [91] and the lncRNA TFAP2A-AS1 [92] were identified as the molecular regulators of miR-584-3p. In detail, the lncRNA LINC00174 is highly expressed in LC tissues, where it is correlated with a shorter OS and with the more aggressive behaviors of cancer cell lines [91]. This aberrant expression induced a downregulation of miR-584-3p and an overexpression of its validated target gene, RPS24. Overall, these data suggested that the downregulation of LINC00174 allowed for the reduction in the malignant phenotypes of LC cells through the modulation of the miR-584-3p/RPS24 axis [91]. In the study of Zhang and colleagues, the lncRNA TFAP2A antisense RNA1 (TFAP2A-AS1) was negatively correlated with the OS of the patients with NSCLC [92]. Mechanistically, TFAP2A-AS1 positively controlled the CDK4 expression through sequestering miR-584-3p, resulting in a boost for the cancer progression [92]. At last, in the study of Li and colleagues, miRNA screening was conducted to identify miRNA correlated with the prognosis of LUAD patients. Of the 209-variance expression identified in the screening analysis, miR-584-3p is one of the miRNAs downregulated in cancer tissues and associated with the overall survival rate [90]. 

Interestingly, miR-584-3p is downregulated in GC tissues and cell lines, where it induces a transcriptional repression of MMP-14 by targeting its promoter, resulting in an arrest of cell proliferation, metastasis and angiogenesis in vitro and in vivo [93].

Finally, via a high-throughput approach, it was found that the Metformin, a drug capable of hindering the aggressiveness of melanoma cells, increased the miR-584-3p expression in these cells. These data were further confirmed through functional assays, which provided evidence that miR-584-3p negatively regulates cell growth through silencing SCAMP3, a protein carrier altered in several cancers [94]. 

## 4. Circulating Level of miR-584-5p in Cancer Patients

In addition to the role of miRNA as a potential anti-cancer agent, increasing evidence revealed the contribution of miRNAs as promising circulating diagnostic and prognostic tools that could improve diagnostic accuracy. To date, studies pertaining to miR-584-3p as a biomarker are not available as of yet; thus, in this section, the tumor biomarker function is discussed exclusively for miR-584-5p, and the results are reported in Table 8.

In LC, two independent studies investigated the potential application of circulating miR-584-5p as a biomarker. From this research, it emerged that miR-584-5p is upregulated in blood obtained from LUAD patients at the diagnosis compared to healthy controls [54,95].

**Table 8:** Circulating level of miR-584-5p as a potential biomarker in human cancer.

Cancer	Sample	Expression	Biomarker Utility	Patients Enrolled in the Study	Technique	Year	Ref.
LA	Serum	Up	Diagnosis	170 LA vs. 170 HC	Exiqon miRNA q-PCR panel (screening) q-RT-PCR (validation)	2020	[54]
	Plasma	Up	Diagnosis	141 LA vs. 124 HC	Exiqon miRNA q-PCR panel (screening) q-RT-PCR (validation)	2017	[95]
CRC	Plasma	Up	Diagnosis	35 CRC vs. 6 HC	NGS (screening) dd-RT-PCR (validation)	2020	[49]
ESCC	Plasma	Up	Diagnosis	178 ESCC vs. 20 HC	Exiqon miRNA q-PCR panel (screening) q-RT-PCR (validation)	2017	[48]
HCC	Plasma	Up	Diagnosis	51 HCC vs. 38 non-HCC	miRNA-seq	2020	[96]
NPC	Plasma	Up	Predictive	17 NPC (before and after therapy)	miRNA PCR plate	2022	[97]

CRC: colorectal cancer; dd-RT-PCR: droplet digital RT-PCR; ESCC: esophageal squamous cell carcinoma; HC: healthy controls; HCC: hepatocellular carcinoma; LA: lung adenocarcinoma; NGS: next-generation sequencing; NPC: nasopharyngeal carcinoma.

The overexpression of the circulating miR-584-5p level has also been revealed in plasma isolated from CRC patients [43] and from ESCC patients, suggesting its potential role as a non-invasive biomarker for the early detection of these tumors [48]. Notably, the higher expression of miR-584-5p occurred not only in the plasma but also in the cancer tissues and exosomes of ESCC patients [48].

In HCC, the circulating level of exosomal-miR-584-5p, in combination with other circulating miRNAs, is able to discriminate with a high sensitivity and specificity the HCC patients from subjects with liver cirrhosis or those infected by hepatitis B virus [96]. 

Finally, a recent study revealed that miR-584-5p is significantly higher in nasopharyngeal carcinoma (NPC) patients who, after treatment with cisplatin and radiotherapy, presented a complete response, suggesting the utility of this miRNA as a predictive biomarker [97].

## 5. Advantages and Drawbacks of the miR-584 Family 

Here, we have interrogated the literature to provide a full landscape on the potential role of the miR-584 family regarding tumor characteristics. In this scenario, our research revealed the rising significance of this family in the regulation of cancer development and progression. 

The advantage of our research is due to the insights given into downstream molecular mechanisms and potential target genes of miR-584-5p. In this scenario, from our in-depth analysis, we found that miR-584-5p modulates the expression of important target genes such as MMP-14, ROCK1 and YAP1, influencing multiple cancer mechanisms, including drug resistance, motility, angiogenesis and cell death.

Regarding miR-584-3p, we have also highlighted that few data emerged from our research, thus suggesting the growing interest mainly in miR-584-5p.

Notwithstanding the growing interest on this topic, there are limitations of our study and also of the other studies mentioned in this comprehensive review that hinder the employment of the miR-584 family in RNA-based therapies and also as a diagnostic tool. First, even if, in this review, 64 articles regarding the miR-584 family are mentioned and discussed, as this is a narrative review, a strong methodology and a quality check are not included. 

Concerning the studies mentioned in our work, in some types of cancers, miR-584-5p appears to be both overexpressed and downregulated. Several conditions, such as the source (blood or cancer tissues), clinicopathological characteristics and method of miRNA collection, could be a possible explanation for these discrepancies.

For example, the demographic cohorts and the small number of cancer patients analyzed in several studies could impair the results, since age, gender, weight, race and lifestyle represent important confounding factors that could influence the endogenous level of miRNAs both in healthy subjects and cancer patients. 

In addition to this, the heterogeneity between cancer patients and also within each individual could be responsible for the variable level of miRNA expression and, consequently, hinder the utility of the miRNA as a diagnostic tool. On the other hand, the intratumor heterogeneity expression is clear in several cancer tissues and could be responsible for altered miRNA expression signatures in distinct regions of the same cancer. 

Another limitation that emerged from our analysis is the great variability of the analytical methodologies used to detect miRNAs expression in tumor tissues and blood. Briefly, the lack of the utility of miRNAs as biomarkers could be due to the absence of standardized protocols between the laboratories on the RNA processing, detection and storage, which hinders clinical validation and often leads to a potential wrong interpretation and to a difficulty in defining specific parameters and cut-offs.

Moreover, in many studies, the potential downstream targets of miR-584-5p and of -3p were not thoroughly investigated, since the luciferase activity assay is often missing. Lasty, the majority of the research relies on the examination of miR-584 family expression and function in cellular models, which is not representative of interactions between cells or of the interactions between cancer cells and the component of the extracellular matrix, being static, and, thus, could not be the same as an in vivo human model.

## 6. Conclusions

In this comprehensive review, we investigated the expression and the molecular signaling pathways associated with the dysregulation of the miR-584 family in human cancer. 

From our literature research, 64 articles on this topic have emerged. Among these articles, only nine of them are focused on miR-584-3p, suggesting a greater interest in miR-584-5p in oncological research. In this scenario, several mechanisms are responsible for the aberrant expression of these miRNAs. For example, several studies unveiled that lncRNAs are implicated in the aberrant regulation of miR-584-5p expression in several cancers, such as bladder [23], NSCLC [56], HCC [69] and NB [79,80]. Likewise, other lncRNAs are identified as the molecular regulators of miR-584-3p expression in CRC [86], HCC [87] and LC [91,92].

Moreover, our research reveals that, through targeting several downstream target genes, miR-584-5p is able to regulate several hallmarks of cancer such as proliferation, motility and the drug response. Overall, these data suggest the potential application of this miRNA as an RNA-based therapy. However, many problems still persist today in the use of miRNAs in clinical practice, mainly correlated with the delivery systems and their potential side effects. In this field, the challenges of RNA-based therapies can be addressed thanks to the accelerated development of nanotechnology that has the aim of using nanocarriers capable of packaging small RNA oligomers that are chemically synthesized as specific miRNA mimics or antisense miRNA inhibitors, hindering their rapid degradation in the extracellular environment. The growing interest in miRNAs as therapeutic agents is also confirmed by the great number of clinical trials, ongoing or completed, that have been deposited in the Clinical Trials Database (http://www.ClinicalTrials.gov, accessed on 17 June 2024). However, the majority of these studies have not yet passed phase III clinical trials, and the majority are currently only in phase I.

Finally, other studies revealed that another promising application of miR-584-5p is its employment as a possible diagnostic tool. Indeed, the circulating miR-584-5p level was upregulated in the peripheral blood of LC [54,95], CRC [43], ESCC [48], HCC [96] and NPC [97] patients, suggesting that its easy detectability can improve the diagnostic accuracy in clinical practice.

In conclusion, this narrative review ideally offers, for the first time, a concise and comprehensive summary of the miR-584 family function in cancer, offering insight for further research.

## Data Availability

Not applicable.
